# The Moderating Role of Cortisol and Negative Emotionality in the Effects of Classroom Size and Window View on Young Children’s Executive Functions

**DOI:** 10.3390/bs14010018

**Published:** 2023-12-25

**Authors:** Kijoo Cha

**Affiliations:** Department of Early Childhood Education, Gachon University, Seongnam-si 13120, Republic of Korea; kijoocha@gachon.ac.kr

**Keywords:** differential susceptibility, preschool, executive functions, classroom size, window view

## Abstract

This study probed how baseline cortisol (BC), negative emotionality (NE), and environmental facets—classroom size and window view—interact to affect executive function (EF) in preschoolers using virtual reality (VR). In a cohort of 144 children aged 61–85 months, BC levels were measured by saliva assays and NE by parental surveys. Participants completed computerized EF assessments both pre- and post-exposure to one of four VR conditions, which varied by classroom size (large vs. small) and window view (natural vs. built). Due to missing data and outlier responses, three children were removed from the analyses. Regression analyses, accounting for initial EFs, revealed that higher BC was significantly associated with better Digit-span task scores in the nature view, while lower BC correlated with improved performance in the built view. With regard to classroom size, children with varying levels of NE benefitted from the large classroom environment, as evidenced by marginally significant improvements on the Corsi block task. However, higher NE children outperformed their lower NE peers in the large classroom, while a trend inverted in the small classroom context. The findings illuminate how the physical components of preschool environments may interact with children’s physiological reactivity, potentially influencing the development of working memory.

## 1. Introduction

Previous research has highlighted the substantial influence of inhabited spaces, where significant time is spent, on individuals’ cognition, emotions, stress levels, and recovery processes. These studies predominantly centered around long-duration habitats like hospital rooms [1,2], workplaces [3,4], and educational institutions, including school buildings and classrooms [5,6,7,8]. However, the majority of these studies have predominantly focused on older populations, including adolescents, college students, and adults, with fewer investigations involving elementary-level students or younger populations. This leaves a notable research gap in exploring the experimental impacts of specific spatial elements on preschool-aged children. 

In the context of early childhood education (ECE) environments, prior studies have largely concentrated on evaluating the overarching quality of physical surroundings and their subsequent correlation with child development [9]. While there are some studies exploring the effects of specific spatial elements in ECE settings—such as the influence of ceiling height and wall color on children’s cooperative behaviors [10], or the impact of spatial arrangement of play areas on prosocial behaviors [11]—the prevailing emphasis on overall quality tends to overshadow the subtle yet significant roles that individual spatial components may play in developmental processes. Also, there is a noted absence of research considering the role and influence of individuals’ differential susceptibilities concerning the impact of the *physical* environment, in spite of the acknowledged potential for varied individual sensitivities to external stimuli [12,13].

In an endeavor to bridge this gap, this study employs VR technology to investigate how various environmental factors, like classroom size and window views, differentially impact young children’s executive functions in alignment with their physiological reactivities to spatial components. This approach seeks to elucidate the interplay between specific environmental aspects and individual susceptibilities in relation to children’s executive functions.

### 1.1. Influence of Nature View and Classroom Size on Individuals’ Cognitions

Studies examining the influence of spatial elements on cognitive performance frequently focus on natural aspects as a significant variable. These research efforts predominantly unveil the positive impacts of exposure to natural features within spaces, such as views of nature through windows or the incorporation of natural elements like indoor plants and images of natural scenery, on individuals’ cognitive and emotional functioning [3,4,14,15,16,17,18]. Grounded in two pivotal theories, the Stress Reduction Theory (SRT) [1] and the Attention Restoration Theory (ART) [16,19], these studies emphasize the multiple benefits provided by nature. The SRT suggests that natural environments provide individuals with a sense of tranquility, aiding in emotional recovery, stress reduction, and promoting relaxation, thus modulating physiological stress responses within individuals. Conversely, the ART underscores the cognitive advantages bestowed by nature, highlighting nature’s role in fostering a gentle intrigue and wonder, described as “soft fascination”. This allows individuals to engage with natural elements without necessitating intense mental focus, facilitating an escape from mental exhaustion. This engagement with nature thus contributes to the rejuvenation of attention and cognitive restoration, bolstering overall cognitive performance.

Building upon these theories, numerous studies have underscored the beneficial influence of nature exposure within educational settings, such as schools and classrooms, on enhancing cognitive outcomes related to attention and memory [3,4,14,15,16,17,18]. For instance, a study exploring the effect of classroom window views on high school students revealed that those with exposure to natural views outperformed peers in built-view settings (views of human-made structures like buildings) on working memory tests (Digit Span Forward and Backward) [17]. However, a few studies focusing on elementary school students have reported mixed findings. For example, fourth-grade students, despite being exposed to classrooms with natural scenery, did not showcase a noteworthy enhancement in their attention and concentration test outcomes, relative to their counterparts in a built-view setting [20]. Conversely, students in grades 5–7, in classrooms with indoor plants, demonstrated significantly higher cognitive performance on tests involving attention, reading, writing, and mathematics [21,22].

Some studies have incorporated Virtual Reality (VR) technology in their experiments to analyze the influence of spatial elements since using VR simplifies the process of creating varied spaces endowed with distinct environmental elements, as compared to the complexity involved in physically establishing multiple spaces. For example, the study by [23], which utilized VR spaces to examine cognitive task performance in relation to the presence of windows, external natural views, and the intensity of natural light, was conducted with graduate students. The findings underscored that environments equipped with relatively large windows, facilitating the entry of natural light and enabling external nature observation, were associated with improved performance on attention and memory tasks, such as object tracking and word recall. In another study, space enriched with natural elements in VR exhibited enhanced execution of working memory tasks and heightened tension–relaxation responses [24].

Research concerning the influence of classroom size is notably limited compared to studies focusing on the impact of natural elements in educational settings. While not centered specifically on classroom size or on children, some research suggests that larger spaces, such as those with higher ceilings, promote a more focused and explorative response [25] and that enhanced concentration was associated with more spacious and unrestricted environments [26]. Studies have rarely explored the implications of the physical size of classrooms, with more efforts being directed toward considerations such as the number of students per class [27,28,29]. The prior research predominantly suggests a negative correlation between class size (number of students per class) and academic achievement [27,28]. In the context of ECE settings, limited individual physical space has been linked to diminished social and cooperative interactions between peers, fewer play behaviors involving gross motor movement, and more solitary and parallel play and aggressive behaviors [30,31,32].

A study by [7] utilized VR technology to explore the influence of classroom size on college students’ cognitive functioning. Four virtual classrooms were designed, each varying in window presence and room size—from a basic, narrow, windowless version to a wider version without windows. The participants’ brainwave activity was monitored as they engaged in cognitive tasks within each virtual environment. The findings showed no significant variations in cognitive performance across different VR spaces, as measured through various tests such as Stroop, Digit Span, Benton, Visual Memory, and Arithmetic. However, differences were observed in brainwave activities according to the varying VR classroom conditions.

Upon review, it is evident that despite the varying volume of studies validating the significant influences of natural elements and room sizes, there has been minimal research conducted specifically targeting young children. Moreover, none of these studies have explored whether the impact of external spatial factors could intertwine with individuals’ differential susceptibility to external stimuli to yield different developmental outcomes. 

### 1.2. Cortisol and Negative Emotionality as a Moderator in the Context of Differential Susceptibility

The differential susceptibility theory (DST) is a conceptual framework that proposes that individuals vary in their susceptibility to environmental influences, which can determine their developmental trajectories [33,34]. According to this theory, some individuals are more malleable and responsive to environmental factors, whether they are advantageous or detrimental. In other words, these individuals show heightened sensitivity to both supportive and adverse environments. Contrary to the traditional “diathesis–stress” model assuming that certain individuals are only more vulnerable to negative outcomes in the face of adversity, the DST emphasizes that individuals who are more susceptible to negative experiences might also be more responsive to positive experiences, leading to a wider range of better or worse outcomes based on environmental context [33,34]. The contrastive effects model, also discussed by [33,34], posits that environmental conditions can yield contrasting effects based on individual sensitivity, leading to opposing developmental outcomes. In this framework, while one group of individuals may thrive in a given environment, another group may experience adverse developmental consequences under the same conditions, and vice versa.

Studies on the Differential Susceptibility Theory (DST) examine the interplay of genetic factors, temperamental traits, and biological markers with environmental conditions in shaping development. They focus on physiological reactivity, which is how the body physically responds to external stimuli, including changes in heart rate, hormone levels, and similar physiological indicators [12,35]. In particular, cortisol and negative emotionality have been focal phenotypic markers of physiological reactivity in this realm. In this line of research, studies have explored how individual differences in cortisol reactivity (change in cortisol levels in response to external stimuli, such as stress or a challenge) might moderate the relationship between environmental experiences and developmental outcomes (e.g., [36,37,38,39,40]). Overall, the accumulated evidence on the moderating effects of cortisol reactivity aligns well with the DST, suggesting that individuals exhibiting elevated cortisol reactivity are more susceptible to both enriching and adverse environmental inputs [35,37,39,41], with some findings corresponding to the diathesis-stress model [36,38]. For example, [42] found that consonant with the DST framework, children with heightened cortisol reactivity manifested enhanced outcomes such as improved emotional regulation, peer relations, and school readiness in nurturing environments, while adverse contexts heralded less favorable results.

Although there has been considerable research exploring cortisol reactivity, relatively few studies have focused on individuals’ basal cortisol, which refers to cortisol levels under normal, non-stressful conditions (e.g., [42,43,44,45,46]) as an indicator of vulnerability or susceptibility. With a scarcity of research, existing studies on basal cortisol have reported mixed findings, with both high and low levels associated with increased susceptibility to environmental influences [44,45,46]. For instance, [46] studied aggression in preschoolers and found that children with *lower* basal cortisol exhibited a significantly greater number of aggressive behaviors, when exposed more to peer victimization, compared to their counterparts with high basal cortisol. However, the opposite pattern was observed when they were relatively less victimized. Similarly, [42] found that children with relatively *lower* basal cortisol at 7 and 24 months exhibited better performance on EF tasks at 48 months when spending more hours in center-based childcare, while such relations were not found among those with higher basal cortisol. In contrast, another study found that boys with *higher* levels of baseline cortisol were more susceptible to their fathers’ parenting practices: they displayed varying levels of aggressive behaviors that were positively related to the degree of authoritarian parenting exhibited by their fathers [45].

These findings, although sourced from a limited number of existing studies, underscore the significant interaction effects of basal cortisol, indicating that both high and low baseline levels may serve as moderating factors, yet in divergent directions. Consequently, further investigation is warranted to elucidate the circumstances under which these varying levels of basal cortisol function as either vulnerability or susceptibility factors, impacting distinct developmental domains such as socio-emotional versus cognitive development.

Next, it has been posited that negative emotionality, defined as a temperament trait marked by a higher intensity and frequency of negative emotions such as sadness, anger, and fear, may act as a moderator in the interactions between individuals and their environment [12,35,47]. The variability in negative emotionality, considered another marker of physiological reactivity, is believed to be influenced by specific genes that determine how individuals respond to environmental stimuli (e.g., 5-HTTLPR, DRD4) [48,49]. These particular genes are understood to be involved in regulating individuals’ emotions and moods, as well as attentional and motivational processes, and stress responses, which in turn contribute to a range of adjustment or developmental outcomes (e.g., ADHD, depressive symptoms, substance use) [12]. Indeed, prior studies delving into the moderating role of children’s negative emotionality have reported supporting evidence of the DST: children relatively higher in that temperamental trait exhibited relatively better attachment to parents, higher socio-emotional and cognitive development, including executive functions, in more favorable surroundings, while faring worse in less favorable environments [12,50,51,52,53]. 

Despite a wealth of research testing the DST and the diathesis–stress model, previous research has predominantly focused on the influence of social environments, such as parenting, educational practices, and interpersonal relationships, on individuals’ development and well-being, often overlooking the potential impact of physical environmental conditions. Notably, there appears to be a lack of studies exploring the interplay between spatial elements of physical environments and individuals’ physiological markers like cortisol and negative emotionality in the context of the DST. Drawing on existing findings, it is plausible to hypothesize that children’s basal cortisol and negative emotionality might affect their adaptive responsiveness to changing physical environments as well. In this context, this study seeks to bridge the gap in the literature by investigating whether children’s differential susceptibility, as indicated by initial cortisol levels (prior to VR exposure) and negative emotionality, interacts with classroom conditions to influence post-performance on tasks measuring executive functions (EFs).

### 1.3. Research Questions and Hypotheses

To scrutinize the variations in children’s EF performance in relation to their sensitivity (i.e., basal cortisol levels and negative emotionality) and different virtual reality (VR) conditions (window view and classroom size), this study posits the following research questions (RQs):

RQ1. Does children’s *baseline cortisol* moderate the relationship between children’s EF performance and VR conditions (i.e., classroom size and window view conditions)? 

RQ2. Does children’s *negative emotionality* moderate the relationship between children’s EF performance and VR conditions (i.e., classroom size and window view conditions)? 

Based on the existing literature, it was expected that both children’s baseline cortisol levels and negative emotionality would play moderating roles in the relationship between VR conditions and children’s EF performance. Specifically, in regards to basal cortisol, it was hypothesized that both high and low levels would interact with contrasting VR conditions, but in opposite directions. That is, it was anticipated that children with higher basal cortisol levels would perform better in more favorable environments (such as a large room and nature view) due to optimal arousal from the calming effects of each setting. In contrast, these children might experience decreased performance in less favorable conditions (such as a small room and built view) due to increased stress and over-arousal. Conversely, children with lower basal cortisol levels were expected to perform better in less favorable environments, where heightened arousal from mild stress could be beneficial, and show reduced performance in more favorable settings due to the decrease in the already low arousal level. This expectation was based on previous research findings that suggest an inverted U-shaped relationship between cortisol and cognitive performance [42,54]. With respect to negative emotionality, consistent with the DST framework, it was posited that children with higher negative emotionality would outperform those with lower negative emotionality in more favorable conditions, while showing poorer performance in less favorable conditions.

## 2. Methods

The dataset utilized in this study was originally compiled to examine the impact of classroom conditions (specifically, classroom size and window view) on executive function (EF) performance and cortisol levels in preschool-aged children using VR technology [55]. It is important to note that the primary intent of the data collection did not include the examination of the moderating effects of cortisol and temperamental characteristics. Consequently, this study represents an exploratory attempt to delve into the under-researched interplay among physiological markers, the physical environment, and EFs among young children.

### 2.1. Participants 

One hundred forty-four pairs of children and their mothers from the capital area of the Republic of Korea were enlisted to participate in the study. The inclusion criteria required children to (1) be without a history of neurological or mental health disorders such as schizophrenia, ADHD, and epilepsy, and have no family members with such conditions; (2) have no history of medication for any unrelated illnesses; (3) have no color blindness and possess a corrected vision of 0.8 or above; (4) be either five or six years old at the time of participation, meaning they were born in 2015 or 2016; and (5) have previous exposure to VR without any unusual reactions. For safety considerations, only five- or six-year-olds in the final year of pre-primary education with prior VR experience were selected. Recruitment was facilitated through the dissemination of flyers on websites frequently visited by mothers of preschool-aged children. Participants’ ages ranged from 61 to 85 months (average age = 74.62, SD = 4.87), with the sample comprising roughly 48.6% males (70 boys and 74 girls). Each participating parent–child duo was compensated with around $50. During participation, children underwent saliva collection, executive function (EF) testing, and a VR experience, while parents filled out questionnaires related to family socio-demographics and the children’s temperaments.

### 2.2. Procedures

#### 2.2.1. Design of VR Classrooms

**Figure 1:**
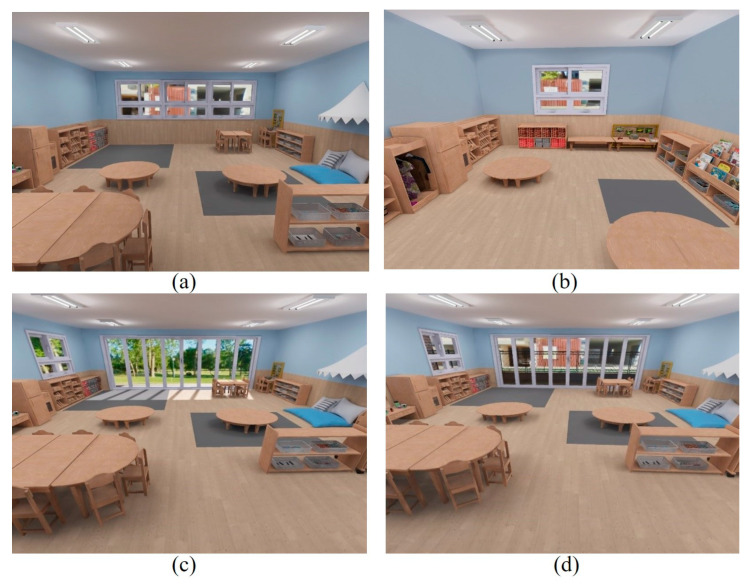
Two-dimensional captures of the four VR preschool classrooms: (**a**) large classroom; (**b**) small classroom; (**c**) nature view; (**d**) built view (Source: [55]).

Four distinct virtual classroom conditions were designed to evaluate two key treatment variables: room size and window view. Variations were made to create two diverse versions of each variable, resulting in a total of four unique virtual classroom environments. First, as for the classroom size condition, the larger classroom dimensions were standardized at 2.6 m (H) × 8.9 m (W) × 7 m (D), allocating 3.1 m^2^ per child based on a presumed class size of 20. This is slightly above the OECD’s average minimum space standard for each child in ECE settings, which stands at 2.9 m^2^ for kindergartens/preschools [56]. Conversely, the smaller classroom, with dimensions of 2.6 m (H) × 5.0 m (W) × 4.0 m (D), allocated merely 1 m^2^ per child, equating to about one third of the larger classroom’s size. The large classroom was created as shown in Figure 1a, and the small classroom was designed as depicted in Figure 1b. Next, regarding the window view condition, a nature view, that is, a classroom with views of a natural landscape such as grass fields and trees (Figure 1c), and a built view, a classroom with views of human-made structures like concrete buildings and streets (Figure 1d), were virtually created. 

To maintain consistency across the different virtual classrooms, all factors other than the treatment variables (room size and window view) were held constant. A notable distinction was that the smaller classroom could not accommodate as many furniture items or play materials compared to the larger room. To avoid influencing the children’s responses, identical types and quantities of essential materials and activity areas were maintained across classrooms. Only non-essential items, mainly peripheral furniture like chairs, were minimized in the smaller classroom version.

#### 2.2.2. Experimental Procedures 

Parents, mostly mothers, registered for participation through online Google Forms. They were first categorized based on the gender of their children and then randomly allocated to one of the four virtual environments. Detailed information regarding the study was communicated to the parents via phone, where aspects like the study’s purpose, processes, and compensation were clarified, and laboratory visits were scheduled subsequently. A reminder, along with specific instructions such as avoiding certain activities that could affect saliva and cortisol analysis (like chewing gum or eating), was sent to the participants a day before the lab visit. The experiments were meticulously timed to account for the natural variations in cortisol levels throughout the day, conducting them consistently at 10 a.m. (classroom size condition) and 2 p.m. (window view condition). Data collection was carried out during the COVID-19 pandemic, necessitating special health precautions. All participants were required to wear masks during the entirety of the experimental procedures to ensure safety. Moreover, the laboratory’s environment where the experiments took place was carefully regulated and maintained, keeping temperature and humidity levels consistent throughout the period of data collection. The assigned conditions were maintained confidentially to prevent any bias or influence on the study’s outcome.

Upon arrival at the university laboratory, a comprehensive briefing was given to the parent–child duos, ensuring clarity and consent regarding the participation in the experimental procedures. Following this, the pairs were guided to the experimental room where they were given five minutes to acclimatize and navigate freely. A research assistant then explained to the child that their mother would return to the waiting room, with the door left half open if they preferred it that way. The mother, while in the waiting room, completed necessary consent forms and questionnaires pertaining to familial socio-demographics and the child’s temperament.

After the mother exited the room, a research assistant gathered saliva samples from the child. The child was then escorted to a computer situated in a corner of the room to undertake computer-based EF tests, which lasted about 15 min. Following the preliminary tests, the child was directed to the middle of the room where a broad square was marked on the floor, indicating a space where the child could move freely while immersed in a virtual reality (VR) environment, donning a VR headset. In the initial phase of the VR experience, the child was guided to a chair positioned at the square’s center and equipped with the VR headset. Subsequently, they had a minute to explore a non-test VR setting (a hallway), allowing them to acclimate to the VR apparatus and determine whether the experience induced any discomfort or issues.

Subsequently, the child engaged with the VR classroom for a duration of ten minutes. Considering the challenge for children to remain seated continuously for ten minutes, they were permitted to move around during the latter five minutes, following an initial five minutes of sitting and observing the room. In previous VR studies involving adults, participants typically spent between four to ten minutes in the experience [7,18,23]. This study, however, allocated a ten-minute exposure time, based on the rationale that children might require more time to navigate and acclimate to a novel virtual setting, and might also desire a longer duration due to the newness of the VR environment.

Once the VR experience concluded, post-test computer-based EF (executive function) tests were administered, and another saliva sample from the child was collected. To mitigate any potential practice effects, minor modifications were made to the content of the post-tests, ensuring the difficulty level remained consistent. For example, changes were made in the digits used in the Digit Span test, and substitutions were made in target objects in other tests. The completion of the entire procedure took no more than sixty minutes.

### 2.3. Measures

#### 2.3.1. Socio-Demographic Information

Data regarding the educational background of the parents were gathered, with respondents selecting from various options: (1) completion of middle school, (2) completion of high school, (3) graduation from a 2- or 3-year college, (4) graduation from a 4-year college, and (5) attainment of graduate-level degrees. Additionally, information pertaining to the monthly family income was also collected, categorized into several brackets ranging from (1) under $999 to (11) over $10,000, with intermediate categories in increments of $1000 (e.g., (2) $1000–$1999, (3) $2000–$2999, and so on).

#### 2.3.2. Negative Emotionality

Information on children’s negative emotionality was collected through parents’ responses to the Children’s Behavior Questionnaire Short Form (CBQ-SF) consisting of three subscales: surgency, negative emotionality, and effortful control [57]. Twelve items on the negative emotionality were assessed using a seven-point Likert scale (Cronbach’s alpha = 0.66). Respondents could choose a value between 1 (indicating that a statement is ‘extremely untrue of my child’) and 7 (signifying that a statement is ‘extremely true of my child’). An additional ‘not applicable’ option was also available for selection. To capture the essence of the construct more accurately, a composite factor score for negative emotionality was calculated, thus being mean centered for the analyses as other variables were.

#### 2.3.3. Executive Functions 

Executive functions are generally understood to encompass working memory (i.e., the ability to hold and manipulate information over short periods), inhibitory control (i.e., the ability to control impulses and resist distractions), and cognitive flexibility (i.e., the ability to switch between different concepts or think about multiple concepts simultaneously) [58]. However, this study measured only two of these three major components: working memory and cognitive flexibility. This focus was aligned with previous studies that predominantly examined changes in memory and attention capacities within the ART and SRT frameworks. Additionally, this study was not originally designed to investigate the interplay among the variables as tested here; thus, the inhibitory control component was not included. That said, three well-established, computer-based EF tasks sensitive to developmental variations, were employed to assess attentional control and multiple forms of working memory (e.g., [59]). The Digit Span Forward Task involved children listening to a series of randomly presented digits and then repeating them orally, assessing their verbal working memory and attention. The complexity of the sequences gradually increased, determining the children’s Digit Span scores based on the longest sequence they could correctly reproduce (from two to seven) [60]. In the Corsi block-tapping task, children were assessed on visuo-spatial working memory and attention. They were required to replicate the sequence of illuminated blocks on a touchscreen, with the sequence’s complexity incrementing upon successful replication. The task duration was contingent on the children’s ability to reproduce the sequences correctly. The children’s Corsi spans or *block spans* (defined as the longest sequence a participant was able to reproduce via tapping, ranging from 0 to 9), were calculated and used in the analysis. The Dimensional Change Card Sorting (DCCS) Task was used to evaluate attentional flexibility [61]. The border version was administered since children in the Republic of Korea exhibited ceiling performance on the DCCS task [62] and the study participants were all five- to six-year-olds at the point of measurement. Scores on the DCCS ranged from 0 to 36, with each trial scored as 0 for incorrect and 1 for correct. The analysis included 12 trials each for color, shape, and border, summing up to a total score.

#### 2.3.4. Cortisol

While basal or baseline cortisol is commonly measured at least twice to ensure measurement reliability, this study utilized a single measurement of cortisol levels taken before the provision of VR stimuli, as the data was not initially collected with the intention of testing the moderating effects of basal cortisol. Although another cortisol level was measured post-VR exposure, this data could not be utilized in the analysis. This was because each VR condition elicited stress responses in opposite directions: the nature view and large classroom conditions likely facilitated stress relaxation, while the built view and small classroom conditions presumably induced stress or at least did not promote relaxation. Despite the limitation inherent in using a single measurement in this study, a strong correlation between the pre- and post-cortisol levels (*r* = 0.76, *p* < 0.001) in the current study supports the reliability of the initial cortisol measurement.

### 2.4. Analyses

Before diving into the research questions, a preliminary analysis was carried out to understand the descriptive statistics and relationships between key variables. In this phase, it was observed that two children had exceptionally high cortisol levels (pre-level: M = 26.57 nmol/L; post-level: M = 33.37 nmol/L), compared to the rest of the sample (pre-level: M = 6.48 nmol/L; post-level: M = 5.82 nmol/L), with a marked and unusual *increase* in post-measurement levels against a normal pattern of diurnal change in cortisol. These levels were significantly elevated relative to existing benchmarks from previous research, such as the findings by [63]. Furthermore, the saliva sample from one child was missing. Consequently, these three children, consisting of one boy and two girls, were omitted from the subsequent analyses. This led to a total of 141 children being analyzed, with distinctions made based on the specific conditions and parameters of the room size and window view in the experiment.

To explore the potential moderating influence of children’s pre-cortisol levels on the relationships between VR conditions and post-EF scores, multiple regression analyses including an interaction term between children’s pre-cortisol level and VR conditions were conducted, with all variables being mean-centered [64]. Children’s pre-EF scores were controlled in the analyses as a covariate to account for initial differences, while socio-demographic variables (family income and parental education) were not controlled since none of these variables was correlated with EF scores (Table 1). In fact, the model fit indices (r-squared and adjusted r-squared) dropped when included.

When significant interaction effects were detected, follow-up analyses were conducted to determine at which levels of a moderator variable—1 SD above the mean, around the mean, and 1 SD below the mean—significant interaction effects were present [64,65]. These follow-up analyses were executed by performing regression analyses that included the dummy-coded variables of the three levels, as well as the interaction terms between each dummy variable and the VR condition.

## 3. Results

### 3.1. Correlational Analysis among Main Study Variables

Correlational analyses among the main study variables are presented in Table 1. Negative emotionality was correlated with none of the children’s pre- and post-EF scores. The children’s pre- and post-test scores showed *moderate to strong* correlations within the respective tasks: Digit Span (*r* = 0.53, *p* < 0.001), Corsi block (*r* = 0.49, *p* < 0.001), and DCCS (*r* = 0.43, *p* < 0.001) (Table 1). Pre- and post-cortisol levels were also strongly correlated (*r* = 0.76, *p* < 0.001). As there were only weak and sporadic correlations among the EF scores across the tasks, the respective tasks were analyzed separately in the main analyses. Minimal and weakly significant correlations were observed between the Corsi-block task and both the Digit Span and DCCS. No notable correlations were found between EFs and cortisol levels. Apart from a slight correlation between the pre-digit-span scores and family income (r = 0.17, *p* < 0.05), there was no significant relationship between children’s EF scores and factors like family income or parents’ level of education. Descriptive data regarding the children’s executive functions and cortisol levels, segmented by experimental conditions, are displayed in Table 2.

### 3.2. Moderating Effects of Children’s Baseline Cortisol 

**Figure 2:**
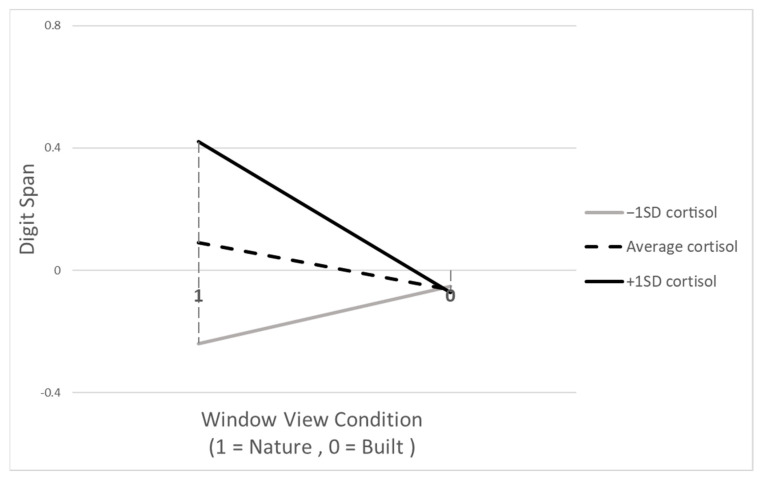
Moderating effects of baseline cortisol: Digit-span score by window view (nature vs. built). Note: In the analyses, the Digit-span score was mean centered; thus, a value of zero corresponds to the mean score.

Results from regression analyses concerning baseline cortisol as a moderator, with executive function (EF) scores as outcome variables, are presented in Table 3. Regression analyses uncovered significant interaction effects in the model exploring the relationship between the nature view condition and the post-digit-span score, when controlling for the pre-digit-span score (refer to Model D in Table 3). As per Model D, children’s baseline cortisol appeared to interact with the window view condition (nature vs. built) in predicting the post-digit-span score. Figure 2 visually illustrates this finding. Children with cortisol levels one standard deviation above the group average demonstrated enhanced performance on the Digit-span task when exposed to the nature view condition. However, their performance regressed to the group’s mean level under the built view condition. Conversely, children whose baseline cortisol was one standard deviation below the group mean exhibited lower-than-average performance in the nature view condition, yet their performance rose to the group mean under the built view condition. Regarding the other models, neither main effects of the VR condition (classroom size and window view) nor interaction effects were detected except for Model C: children in the large classroom condition were found to exhibit a marginally significant higher performance on DCCS compared to the counterparts in the small classroom condition.

Follow-up analyses pinpointed the presence of significant interaction effects between groups below (−1SD) and above the mean (+1SD). The interaction effect slopes displayed notable differences, proving to be significantly diverse (*t* = 2.29, *p* = 0.025). This result supports the hypothesized inverted U-shaped relationship between cognitive performance and basal cortisol as a marker of a greater malleability to the beneficial effects of the natural landscape.

### 3.3. Moderating Effects of Children’s Negative Emotionality

**Figure 3:**
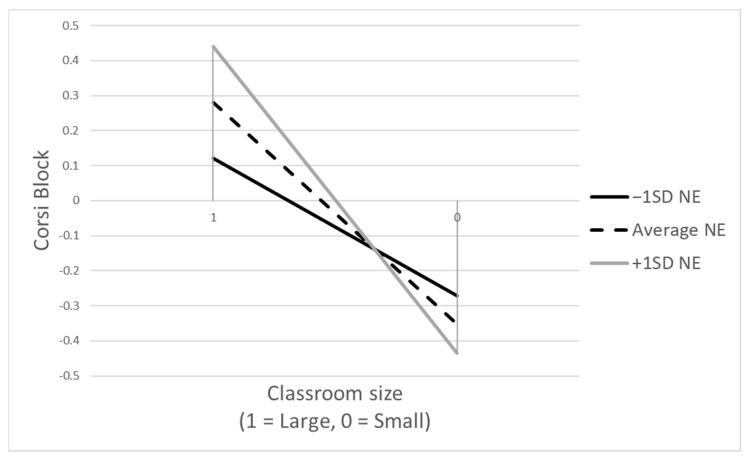
Moderating effects of negative emotionality: Corsi block score by classroom size (large vs. small); Note: NE = negative emotionality. In the analyses, the Corsi block score was mean centered; thus, a value of zero corresponds to the mean score.

In models assessing negative emotionality as a moderating variable (refer to Table 4), a *marginally* significant interaction was observed between negative emotionality and the post-Corsi block scores, when accounting for the pre-Corsi block scores as a covariate (see Model B in Table 4). This interaction suggests a *potential* moderation by children’s negative emotionality in the relationship between classroom size conditions (large vs. small) and the Corsi block scores. Figure 3 illustrates this outcome. Here, children exhibiting higher negative emotionality (one standard deviation above the mean) demonstrated improved performance in the large classroom condition, while their performance fell below the group mean in the small classroom condition. On the other hand, children with lower negative emotionality (one standard deviation below the mean) exhibited a more modest decline in performance between the two conditions compared to their high negative emotionality (+1SD) counterparts. Notably, the group with lower negative emotionality (−1SD) displayed inferior performance compared to the high negative emotionality group (+1SD) in the large classroom condition, but outperformed them in the small classroom condition. This pattern is consistent with the reduced susceptibility as suggested by the DST. Paying attention to Model C (Table 4), it is also notable that, paralleling the earlier findings with children’s baseline cortisol as a moderator (Table 3), the large classroom condition once more exhibited a significant positive influence on the DCCS scores of children.

Follow-up analyses revealed that significant interaction effects were present between the below-the-mean (−1SD) and above-the-mean groups (+1SD), with the slopes for interaction effects differing at the level of marginal significance (*t* = 1.85, *p* = 0.079).

## 4. Discussion

Prior research on early childhood education (ECE) environments and cognitive development has yielded mixed results, which have been posited to partly arise from children’s varying sensitivity to their surroundings [42,66]. This pioneering study explored how such differential susceptibility, alongside the spatial aspects of physical environments, affects executive functions in children. It found that higher baseline cortisol levels—a physiological reactivity indicator—correlated with better verbal working memory (Digit Span) in the nature-view condition compared to the built-view condition. Similarly, children with more pronounced negative emotionality demonstrated marginally significantly higher performance on visuo-spatial working memory (Corsi block) in the large classroom condition, outperforming those with lower negative emotionality. In contrast, the reverse pattern was observed in the small classroom condition.

Regarding baseline cortisol’s moderation effects, a pattern of *contrastive effects* rather than differential susceptibility was observed [33,34]: children with higher and lower baseline cortisol levels showed opposing patterns of change in their Digit Span scores when comparing nature versus built views. These results seem to relate to the well-documented inverted U-shaped relationship between cortisol and cognitive functions, as hypothesized [54,67,68], where cortisol facilitates cognition by enabling an adequate level of arousal, except at very high levels of production. In other words, moderate cortisol levels are supportive of executive functions (EF) and other cognitive abilities [63,68]. Ref. [63] found that five- and six-year-old children with higher baseline cortisol levels outperformed EF-related tasks, such as attentional control and behavioral inhibition. Ref. [42] also reported that children with relatively lower basal cortisol at 24 months showed improved executive function at 48 months when they spent more hours per week in center-based childcare, which provided greater, yet moderate, stress levels than home settings, likely enhancing cognitive arousal over extended periods.

Indeed, the post hoc examination of pre-to-post cortisol levels in the window view conditions (nature vs. built) generally supported the hypothesized inverted U-shaped patterns. A common trend in line with the natural diurnal rhythm of cortisol was observed, characterized by a decrease from baseline to post-measurement. However, distinct patterns were noted among the different baseline cortisol groups. In the built-view condition, the lower baseline cortisol group (−1SD) (n = 6) showed a marginally significant increase in post-measured cortisol (*t* = −1.86, *p* = 0.06; Pre: 2.75 nmol/L, Post: 4.65 nmol/L), unlike the average group (n = 28), which experienced a significant decrease (*t* = 2.47, *p* = 0.01; pre: 5.77 nmol/L, post: 5.00 nmol/L). The higher group (+1SD) (n = 4) displayed no significant change with persistently high levels of cortisol (*t* = 0.12, *p* = 0.54; pre: 10.86 nmol/L, post: 10.72 nmol/L), potentially indicating a stress response in the built-view environment for both lower and higher cortisol groups. This response could explain the increase in Digit Span scores among the lower cortisol group in the built view, as opposed to their counterparts’ performance in the nature view. Similarly, the diminished Digit Span performance in the higher cortisol group might be linked to the persistent high cortisol levels, possibly interfering with working memory due to over-arousal. In contrast, the nature view condition showed that while the lower cortisol group (−1SD) (n = 6) did not exhibit the expected significant change (*t* = 1.47, *p* = 0.10; pre: 4.28 nmol/L, post: 2.92 nmol/L), both the average (n = 23) (*t* = 3.77, *p* < 0.001; pre: 6.41 nmol/L, post: 5.38 nmol/L) and higher groups (n = 5) (*t* = 3.19, *p =* 0.01; pre: 11.12 nmol/L; post: 9.56 nmol/L) showed a significant decrease in cortisol, suggesting a relaxation effect. This may have contributed to the higher group’s improved Digit Span performance in the nature view compared to their built-view counterparts. However, the interpretation of these findings must be approached with caution, considering the small sample sizes of each cortisol-level group and the overall study.

Children in the present study, raised in middle- to upper-middle-class families without exposure to chronic stressors, exhibited a normal range of cortisol levels, including those in the higher cortisol group. While no official standard range of diurnal cortisol for non-clinical young children exists, the mean cortisol levels in the study’s window view condition (measured at around 2 p.m.) were 2.77 nmol/L (SD = 0.50) for the lower group, 12.12 nmol/L (SD = 1.74) for the higher group, and 6.17 nmol/L (SD = 1.62) for the average. Compared to the values reported by [63] for 5- to 6-year-olds—0.71 µg/dL (=19.59 nmol/L) at approximately 8 a.m. and 0.25 µg/dL (=6.90 nmol/L) at approximately 4:30 p.m.—this study’s results suggest that, while generally within the normal range, the cortisol levels in the lower and higher groups might be considered relatively low and high, respectively. Given that moderate, but not excessive, cortisol levels can facilitate a certain degree of arousal, the post hoc results could explain why children in the lower and higher cortisol groups demonstrated improved and diminished performance, respectively, in the built view condition.

Regarding the association between negative emotionality and Corsi-block performance in different classroom sizes, in alignment with the hypothesized result, negative emotionality was linked to poorer performance in the small room (more stressful) but to better performance in the large room (less stressful), though this result was only marginally significant. This finding supports the differential susceptibility hypothesis, suggesting temperamental reactivity acts as a susceptibility factor [33,34]. It is also consistent with prior studies that have explored how this phenotypic marker of physiological reactivity moderates the relationship between cognitive functioning and social environments [39,41,47]. Furthermore, the observation that varying levels of negative emotionality correspond with different degrees of EF performance supports the notion of *differential susceptibility*, which recognizes a continuum of plasticity, as opposed to a bimodal distribution typified by the dandelion versus orchid child dichotomy [47,69].

This study’s findings suggest that baseline cortisol levels and negative emotionality serve as independent moderators of young children’s cognitive development, in line with the *contrastive effects* and *differential susceptibility* models, respectively [33,34]. This indicates that both factors significantly influence cognition through potentially distinct mechanisms. For instance, a study by [45] found that high basal cortisol levels and negative emotionality had different interactions with a child’s gender and paternal parenting styles in relation to aggressive behaviors. Higher basal cortisol interacted with authoritative parenting among boys, with higher negative emotionality with permissive parenting among girls. The absence of a relationship between negative emotionality and cortisol levels in the current study implies that, although both are related to the hypothalamic–pituitary–adrenal (HPA) axis [70,71], they may operate through distinct pathways. This is supported by findings of no or weak correlations between cortisol levels and temperamental negative emotionality [45,72]. Additionally, while genes like 5-HTTLPR, DRD4, and BDNF are known to influence emotional regulation and stress response, which moderates individuals’ interactions with the environment in relation to cortisol and temperamental characteristics [12,48,49], current understandings of the specific roles of these biological moderators and their interplay remains limited, necessitating further research.

In this study, the primary effects of VR conditions on EF task performance were noted with the Dimensional Change Card Sort (DCCS), which measures attentional flexibility, particularly in varying classroom sizes. This effect was not observed with tasks assessing working memory, such as Digit Span and Corsi block, nor in the window view condition. [55], based on the same dataset, discusses potential reasons why a larger classroom size might beneficially impact DCCS performance. The current study extends [55]’s findings by suggesting that the commonly held belief in the cognitive benefits of natural elements exposure may not uniformly apply. Specifically, exposure to nature might not benefit children requiring stimulation rather than relaxation for enhanced arousal and better working memory performance, as seen in those with low basal cortisol levels. Additionally, the findings cautiously indicate that a smaller, more confined classroom may adversely affect children with higher temperamental difficulty levels to a greater extent. These insights complement [55]’s observations where no significant main effects were noted in the window view condition on children’s EF measures.

Yet, it remains unclear why the current study found moderation effects in working memory (as assessed by Digit Span and Corsi block) but not in cognitive flexibility (measured by the DCCS). This might imply that working memory is more sensitive to environmental influences through individual traits like temperament and basal cortisol, along with related biological mechanisms. However, existing research has not empirically established such relationships. Instead, prior studies highlight the conceptual overlap and functional interdependence between working memory and attention [73,74], suggesting the difficulty in isolating the differential susceptibility of each function to individual differences. Similarly, the distinct impact of window views on verbal working memory and classroom sizes on visuo-spatial working memory poses questions that are yet unanswered by empirical research. Although a review by [75] suggests that natural elements have a low to moderate positive effect on both working memory and cognitive flexibility, the specific underlying mechanisms remain elusive. Neuroimaging studies have revealed common brain regions, like the prefrontal cortex (PFC) and anterior cingulate cortex (ACC), activated during tasks involving working memory and attentional flexibility [76,77]. However, distinctions have also been found, with areas such as the ventrolateral prefrontal cortex being differently engaged in working memory and attention systems [78]. The phonological loop and visuo-spatial sketchpad, responsible for verbal and visuo-spatial working memory, respectively, also suggest neural differentiation [79,80,81]. This intricate interplay warrants further investigation, especially concerning potential domain-specific effects within the framework of differential susceptibility [69].

### Limitations and Future Research

While the present study possesses several merits, including pioneering the examination of physical reactivity’s moderating effects in conjunction with spatial components and focusing on the underrepresented preschool demographic in such research, it is not without its limitations that future studies should aim to address. Firstly, the measurement of children’s basal cortisol levels was conducted at only a single time point. Despite a strong correlation observed between pre- and post-cortisol levels (r = 0.76, *p* < 0.001) bolstering the reliability of the initial readings and despite precedents for using a single cortisol measurement being established (e.g., [82]), subsequent research should employ multiple measurements to ensure greater reliability.

Further investigation into the interplay of physiological reactivity, such as cortisol reactivity, with a wider array of spatial elements, including both classroom size and window view, could offer a more comprehensive understanding of these interactions. While the current study focused on how children’s biological and temperamental characteristics interact with classroom size and window view independently, future research could productively explore their combined effects. Such an approach might reveal significant insights into the potential compensatory effects of these environmental variables when considered together, particularly in relation to children’s developmental outcomes.

An additional limitation of this study is the variation in window sizes between the large and small classrooms, which may have impacted the results. Future research should strive to control for such discrepancies in VR classroom environments to ensure that the observed effects are attributable to the primary variable of interest, rather than to confounding factors. Furthermore, the present study was carried out in a highly urbanized setting. The impact of window views (nature versus built) may vary in rural populations where natural vistas are more common and built views less so. Exploring these differences in future research could be a valuable avenue of investigation.

Next, further research is needed to explore various facets of self-regulation, encompassing both behavioral and emotional regulation, as well as the full spectrum of executive functions, including inhibitory control which was not assessed in the current study. This approach would also enable a more comprehensive assessment of broader developmental outcomes. Additionally, this study’s methodology involved using two distinct groups of children assigned randomly to either experimental or control VR conditions. While initial *t*-test results indicated no significant differences between these groups across main and socio-demographic variables (see [55]), a crossover study design, where the same children experience both conditions, might offer better control for confounding factors that are not the primary variables of interest. Furthermore, enlarging the sample size would be beneficial in increasing the statistical power and decreasing the effects of outliers, thereby enhancing the robustness and generalizability of the study’s findings.

## 5. Conclusions

Despite the wealth of evidence indicating that the physical environment can influence individuals’ cognitive, emotional, and physiological functioning, research into the moderating role of physiological markers of sensitivity to external stimuli within physical settings has been notably absent. This study marks an initial foray into investigating these physiological moderators in early childhood education (ECE) settings, with a particular focus on the effects of window views and classroom size. Given the absence of prior research in this specific area and the exploratory nature of this study, any conclusions should be approached with prudence. Nevertheless, the preliminary findings suggest a potential interplay between certain spatial components and children’s physiological profiles, which may varyingly impact cognitive functioning. This effect seems contingent upon the arousal or relaxation needs triggered by external stimuli. Therefore, future research is critical in establishing a knowledge base that can guide the development of specific recommendations for designing physical environments. Such environments would be tailored to the unique needs of specific populations, particularly young children with varying levels of basal cortisol and negative emotionality, as explored in this study within the context of early childhood education settings.

## Figures and Tables

**Table 1:** Correlation analysis between key variables and socio-demographic factors.

	1	2	3	4	5	6	7	8	9	10	11	12
1. Neg Emo	−	−	−	−	−	−	−	−	−	−	−	−
2. Pre-DS	−0.01	−	−	−	−	−	−	−	−	−	−	−
3. Post-DS	0.05	0.53 ***	−	−	−	−	−	−	−	−	−	−
4. Pre-CB	0.16	0.08	0.11	−	−	−	−	−	−	−	−	−
5. Post-CB	0.01	0.12	0.18 *	0.49 ***	−	−	−	−	−	−	−	−
6. Pre-DCCS	−0.08	0.09	0.03	0.19 *	0.18 *	−	−	−	−	−	−	−
7. Post-DCCS	−0.18	0.06	0.00	0.17 *	0.08	0.43 ***	−	−	−	−	−	−
8. Pre-cortisol	0.13	0.15	0.11	0.02	0.01	0.11	0.00	−		−	−	−
9. Post-cortisol	0.05	0.14	0.07	−0.01	−0.01	0.10	0.02	0.76 ***	−	−	−	−
10. Father Edu	−0.08	0.09	−0.03	0.04	0.01	0.04	0.05	0.07	0.10	−	−	−
11. Mother Edu	0.04	0.16	0.13	−0.01	0.02	−0.02	0.01	0.11	0.08	0.42 ***	−	−
12. Income	−0.11	0.17 *	0.16+	−0.01	0.11	−0.01	−0.00	0.25 **	0.14	0.26 **	0.29 ***	−
13. Child’s sex	0.00	0.00	0.01	−0.02	0.12	−0.06	0.11	0.11	0.03	0.04	0.09	0.20 *

Note.: Neg Emo = negative emotionality (composite factor score); pre- = pre-VR exposure; post- = post-VR exposure; DS = Digit Span, CB = Corsi block; DCCS = dimension change card sorting; Father Edu = father’s education; Mother Edu = mother’s education; income = family income; child’s sex:1 = boys, 2 = girls; * *p ≤* 0.05, ** *p ≤* 0.01, *** *p ≤* 0.001.

**Table 2:** Descriptive statistics for children’s executive functions, cortisol levels, and negative emotionality by experimental conditions (N = 141).

		Room Size		Window View	
		Large (n = 34)	Small (n = 35)	Nature (n = 34)	Built (n = 38)
		M (SD)	M (SD)	M (SD)	M (SD)
Digit Span	Pre	3.67 (0.68)	3.57 (0.88)	3.50 (1.05)	3.26 (1.08)
	Post	3.79 (0.97)	3.65 (0.93)	3.76 (1.13)	3.50 (0.86)
Corsi block	Pre	3.12 (1.45)	3.66 (1.13)	3.18 (1.35)	2.79 (1.21)
	Post	3.29 (1.40)	3.14 (1.42)	3.14 (1.37)	2.71 (1.50)
DCCS	Pre	28.85 (3.70)	29.57 (4.05)	28.26 (4.26)	28.08 (3.67)
	Post	30.68 (3.51)	29.26 (3.57)	29.76 (3.54)	29.32 (3.97)
Cortisol	Pre	7.19 (3.55)	6.47 (2.77)	6.49 (2.83)	5.83 (2.48)
	Post	6.21 (2.45)	5.77 (2.01)	5.81 (2.32)	5.55 (3.01)
Negative Emotionality	-	3.84 (0.65)	3.92 (0.68)	3.74 (0.66)	3.93 (0.69)

Note. M = mean; SD = standard deviation; pre = pre-VR exposure; post = post-VR exposure.

**Table 3:** Regression models predicting post-EFs with interaction between the VR condition and baseline cortisol.

	Classroom Size			Window View		
Outcome Variable	Digit Span (Post)	Corsi Block (Post)	DCCS (Post)	Digit Span (Post)	Corsi Block (Post)	DCCS (Post)
	Model A β (SE)	Model B β (SE)	Model C β (SE)	Model D β (SE)	Model E β (SE)	Model F β (SE)
EF score (pre)	0.47 *** (0.14)	0.54 *** (0.12)	0.34 ** (0.10)	0.56 *** (0.08)	0.57 *** (0.11)	0.48 *** (0.10)
Cortisol (pre)	−0.04 (0.05)	−0.04 (0.08)	0.02 (0.21)	−0.03 (0.05)	0.00 (0.08)	0.17 (0.21)
VR condition	−0.26 (0.52)	−0.49 (0.75)	3.35 ^†^ (1.95)	0.15 (0.46)	0.26 (0.77)	2.57 (2.01)
(Pre-cortisol) × (VR condition)	0.05 (0.07)	0.14 (0.10)	−0.23 (0.27)	0.14 * (0.07)	−0.01 (0.11)	−0.36 (0.30)
R^2^	0.16	0.26	0.20	0.45	0.27	0.26
Adjusted R^2^	0.10	0.22	0.15	0.42	0.23	0.21
F	3.07	5.77	4.08	13.83	6.33	5.92
(df)	(64)	(64)	(64)	(67)	(67)	(67)
*p*	<0.05	<0.001	<0.01	<0.001	<0.001	<0.001

Note. SE: standard error; pre = pre-VR exposure; post = post-VR exposure. VR condition: classroom size (large room = 1; small room = 0) and window view (nature view = 1; built view = 0); ^†^ *p* < 0.10, * *p* < 0.05, ** *p* < 0.01, *** *p* < 0.001.

**Table 4:** Regression models predicting post-EFs with interaction between the VR condition and negative emotionality.

	Classroom Size			Window View		
Outcome Variable	Digit Span (Post)	Corsi Block (Post)	DCCS (Post)	Digit Span (Post)	Corsi Block (Post)	DCCS (Post)
	Model A β (SE)	Model B β (SE)	Model C β (SE)	Model D β (SE)	Model E β (SE)	Model F β (SE)
EF score (Pre)	0.46 **	0.52 ***	0.35 **	0.59 ***	0.56 ***	0.48 ***
NE	0.05	−0.52 ^†^	−0.10	0.20	0.11	0.25
VR condition	0.09	0.49	1.67 *	0.14	0.18	0.26
(NE) × (VR condition)	0.08	0.70 ^†^ (*p* = 0.086)	−0.63	−0.26	−0.37	−0.90
R^2^	0.16	0.27	0.2	0.43	0.29	0.26
Adjusted R^2^	0.11	0.23	0.15	0.39	0.25	0.21
F	3.08	5.97	4.08	12.37	6.8	5.85
(df)	−64	−64	−64	−67	−67	−67
*p*	<0.05	<0.001	<0.01	<0.001	<0.001	<0.001

Note. SE: standard error; pre = pre-VR exposure; post = post-VR exposure; NE = negative emotionality. VR condition: classroom size (large room = 1; small room = 0) and window view (nature view = 1; built view = 0); ^†^ *p* < 0.10, * *p* < 0.05, ** *p* < 0.01, *** *p* < 0.001.

## Data Availability

The data that support the findings of this study are available from the author upon reasonable request.
